# Biosafety and Ecological Assessment of Genetically Engineered and Edited Crops

**DOI:** 10.3390/plants12132551

**Published:** 2023-07-05

**Authors:** Wei Wei, Charles Neal Stewart

**Affiliations:** 1 State Key Laboratory of Vegetation and Environmental Change, Institute of Botany, Chinese Academy of Sciences, Beijing 100093, China; 2 Department of Plant Sciences and Center for Agricultural Synthetic Biology, 112 Plant Biotechnology Building, University of Tennessee, Knoxville, TN 37996, USA

Nearly three decades have passed since the first commercial cultivation of genetically engineered (GE) crops. Even prior to commercialization, there were studies testing the biosafety and ecological risks of the release of GE plants. While we have learned much, and the National Academies of Sciences, Engineering, and Medicine Committee on genetically engineered crops found no substantiated evidence showing foods from GE crops were less safe than foods from non-GE crops [1], concerns and controversial views remain. The vast majority of GE crops cultivated by area are annual row crops that are either resistant to herbicide or produce pesticide or of stacked traits of both herbicide resistance and pesticide production. We have gained much experience growing these crops [1]. Although people are optimistic about the environmental and economic benefits conferred by the adoption of GE crops, there have been some predicted risks that have been actualized. For example, secondary insect pests have replaced the niche of the primary pests after the wide adoption of resistant plants that target and suppress populations of primary insect pests, e.g., the study on GE cotton by Lu et al. [2]. Genetic contamination either by pollen or seed flow in native maize varieties of Mexico has been confirmed [3].

Genes isolated from *Bacillus thuringiensis* (*Bt*) are widely used in the first generation of GE crops and to express various insecticidal Bt proteins in host plants to protect them from insect damage. When insect pests evolved resistance to Bt proteins, new Bt proteins were produced in host plants for pest control [4]. Studies have been performed to evaluate the efficacy of those new Bt strains, or a combination of them, against insect pests. There are two research papers in this Special Issue, titled “Biosafety and Ecological Assessment of Genetically Engineered and Edited Crops”, addressing this concern [5,6].

Once GE plants are released into the environment, they interact with various factors, including those involving food chains and competition at multiple levels (gene, individual, population, community, and ecosystem) through trophic connections, nutrient cycles and energy flows, as well as biogeochemical cycles, in contact with abiotic/biotic elements of soils, water, and above- and underground ecosystems in the receiving environment [7,8,9,10,11,12,13]. For instance, GE plants as primary producers play important roles in the receiving environment to convert light energy or chemical energy into organic compounds, which are used as food for other organisms in natural ecosystems. Herbivorous insect pests feed on GE plants and are then preyed upon and/or parasitized by predators and/or parasitoids, e.g., the work of Wei et al. [14] and Guan et al. [15]. When the plants die and decay, decomposers convert those decaying materials (and other wastes, including dead animals) into inorganic materials in soils that support a new cycle commenced by the growth of new primary producers, such as plants. Plant compounds could also be exuded from the roots into soils and may affect soil organisms [16]. The engineered genes and their products (such as proteins) in GE plants could accumulate at or transfer through different trophic levels and actively participate in natural processes (cycles) in the receiving ecosystems and could cause unintended effects to the exposed organisms (Figure 1).

This Special Issue includes four research papers on the impacts of Bt crops on the arthropod community [15,17,18,19] and two research reports on the effects on soil microbiome [20,21] in the lab and in the field. Generally, no overall significant change was observed in the field studies as the environmental conditions and plant growth stage were likely stronger effects than the engineered status of crop cultivars. Two review papers [16,22] analyzed and discussed the current progress of the impacts of GE crops on soil microbiota.

Similarly, a feeding study in this Special Issue showed the absence of adverse effects of a drought-tolerant GE wheat line to experimental rats compared to its non-GE parent crop [23]. “Omics” technologies are proposed to quantify the differences between GE and non-GE foods to inform regulation strategies [24]. A proteomic case study presented in this Special Issue suggested that no shared change occurred between the two GE oilseed rape (*Brassica napus*) lines transformed by the *Bt Cry1Ac* gene [25]. However, in GE corn, the transformation of the *Bt Cry1Ab* gene may affect plant defenses with plant hormones [26], which could suggest that a potential change in metabolomics could be important [24]. Another important aspect of the biosafety concern for the release of GE crops is transgene escape through pollen or seed flow, which may increase or reduce the fitness of the gene flow recipient plants [27]. One review paper included in this Special Issue proposed and discussed potential approaches to bioconfine transgene flow [28].

While controversial debates on those adverse impacts of the first generation of GE plants using transgenic technology continue and some of those concerns remain unresolved, new breeding tools such as gene editing have been developed and widely employed in scientific research for quantity and quality/nutrition improvement in agriculture and food production [29]. Regarding the fast development of gene-edited crops, this Special Issue also published a review paper on the regulation perspectives of these novel crops [29]. In some countries, this kind of breeding tool may be exempt from the sorts of regulation imposed on GE crops when genetic manipulation does not involve or result in the presence of transgenes. Although there are still concerns regarding the release of gene-edited crops, it is unfortunate that no experimental studies have been reported yet on the biosafety and ecological consequences of the edited crops. However, we believe that the commercially adopted edited crops can benefit from lessons learned from the first generation of engineered crops. Holistic approaches may be helpful to evaluate both the benefits and risks of those GE crops in the view of sustainable agriculture.

In summary, the papers collected in this Special Issue addressed some crucial aspects of the interaction of GE crops with organisms in the environment. Although there is no report on the experimental evaluation of the application of novel breeding tools, such as gene editing, previous works with genetically engineered crops may provide valuable experiences for new gene-edited plants.

## Figures and Tables

**Figure 1:**
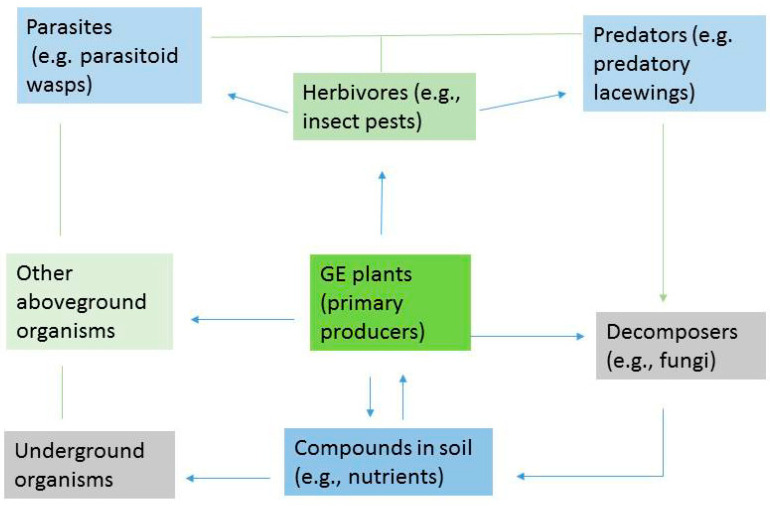
An example of the interaction of genetically engineered (GE) plants with organisms in released environment.
